# Postoperative Management of Portal Vein Arterialization: An Interdisciplinary Institutional Approach

**DOI:** 10.3390/cancers16132459

**Published:** 2024-07-04

**Authors:** Ali Majlesara, Mohammad Golriz, Ali Ramouz, Elias Khajeh, Nastaran Sabetkish, Mark O. Wielpütz, Hugo Rio Tinto, Sepehr Abbasi Dezfouli, Martin Loos, Arianeb Mehrabi, De-Hua Chang

**Affiliations:** 1 Department of General, Visceral and Transplantation Surgery, University of Heidelberg, 69120 Heidelberg, Germany; ali.majlesara@med.uni-heidelberg.de (A.M.); mohammad.golriz@med.uni-heidelberg.de (M.G.); ali.ramouz@med.uni-heidelberg.de (A.R.); elias.khajeh@med.uni-heidelberg.de (E.K.); nastaran.sabetkish@med.uni-heidelberg.de (N.S.); sepehr.abbasidezfouli@med.uni-heidelberg.de (S.A.D.); martin.loos@med.uni-heidelberg.de (M.L.); arianeb.mehrabni@med.uni-heidelberg.de (A.M.); 2 Liver Cancer Center Heidelberg (LCCH), University of Heidelberg, 69120 Heidelberg, Germany; 3 Department of Interventional Radiology, University of Heidelberg, 69120 Heidelberg, Germany; mark.wielpuotz@med.uni-heidelberg.de; 4 Radiology Department, Champalimaud Foundation, 1400-038 Lisbon, Portugal; hugo.riotinto@fundacaochampalimaud.pt; 5 Department of Diagnostic and Interventional Radiology, Heidelberg University Hospital, 69120 Heidelberg, Germany; 6 Department of Radiology, Lucerne Kantonsspital, Spitalstrasse, CH-6000 Lucerne, Switzerland

**Keywords:** hepatic artery, hepatopancreatobiliary surgery, portal vein, portal vein arterialization

## Abstract

**Simple Summary:**

Portal vein arterialization (PVA) is a critical surgical intervention employed when hepatic artery blood flow restoration is unattainable. By redirecting arterial blood to the portal vein, PVA ensures adequate oxygenation of the liver. However, this procedure can induce complications such as portal hypertension and thrombosis if not meticulously managed. The absence of standardized postoperative care protocols exacerbates patient risks. In response, we have developed a standard operating procedure (SOP) tailored for radiologists. This modified SOP is designed to minimize complications and enhance patient outcomes by providing clear, consistent guidelines for post-surgical care, thereby fostering improved interdisciplinary collaboration. Our initiative aims to standardize PVA care, ensuring safer and more effective outcomes for patients requiring this procedure.

**Abstract:**

Portal vein arterialization (PVA) is a surgical procedure that plays a crucial role in hepatic vascular salvage when hepatic artery flow restoration remains elusive. Dedicated diagnostic vascular imaging and the timely management of PVA shunts are paramount to preventing complications, such as portal hypertension and thrombosis. Regrettably, a lack of standardized postoperative management protocols for PVA has increased morbidity and mortality rates post-procedure. In response to this challenge, we developed a PVA standard operating procedure (SOP) tailored to the needs of interventional radiologists. This SOP is designed to harmonize postoperative care, fostering scientific comparability across cases. This concise brief report aims to offer radiologists valuable insights into the PVA technique and considerations for post-PVA care and foster effective interdisciplinary collaboration.

## 1. Introduction

The hepatic artery is responsible for supplying 25% of the liver’s blood flow and providing 50% of its oxygen, particularly to the biliary tract [1,2]. The remaining blood flow to the liver is delivered through the portal vein (PV) [3,4]. However, in cases where the flow supplied by the hepatic artery cannot be restored, portal vein arterialization (PVA) emerges as a potential salvage option [5,6,7,8].

PVA, initially described by Sheil et al. in 1989, has become a widely used technique in the context of liver transplantation [9]. Bonnet et al. suggested that patients with pre-existing diffuse PV thrombosis or those encountering PV thrombosis following orthotopic liver transplantation particularly benefit from this surgical approach [10]. Furthermore, when the right hepatic artery subdivides early into its sectorial branches or in situations where reconstruction of the right hepatic artery is unfeasible because of its caliber, PVA may offer an effective solution [11,12,13,14,15,16,17,18,19].

Despite the potential promise of PVA as an emergency intervention to mitigate hypoxic-ischaemic damage across all liver cells, it is not without its challenges. Patients with hepatic artery thrombosis following liver transplantation who undergo PVA may experience persistent portal hypertension, which could contribute to the development of liver fibrosis because of excessive arterialization of the liver [20]. Moreover, reports of aneurysmal dilatation of the PV and its intrahepatic branches, occurrence of right-sided heart failure, and consequences of portal hypertension have raised concerns regarding the postoperative phase [21]. Thus, effective postoperative care is paramount for patients receiving prompt attention and who have mitigating complications.

In this context, we introduced a modified standardized operating procedure (SOP) based on our comprehensive literature review of PVA outcomes [7] as well as the clinical outcome of a cohort of patients who underwent PVA. We aimed to enhance the critical postoperative management of patients undergoing PVA during hepatopancreatobiliary (HPB) procedures.

## 2. Methods and Results

**Figure 1:**
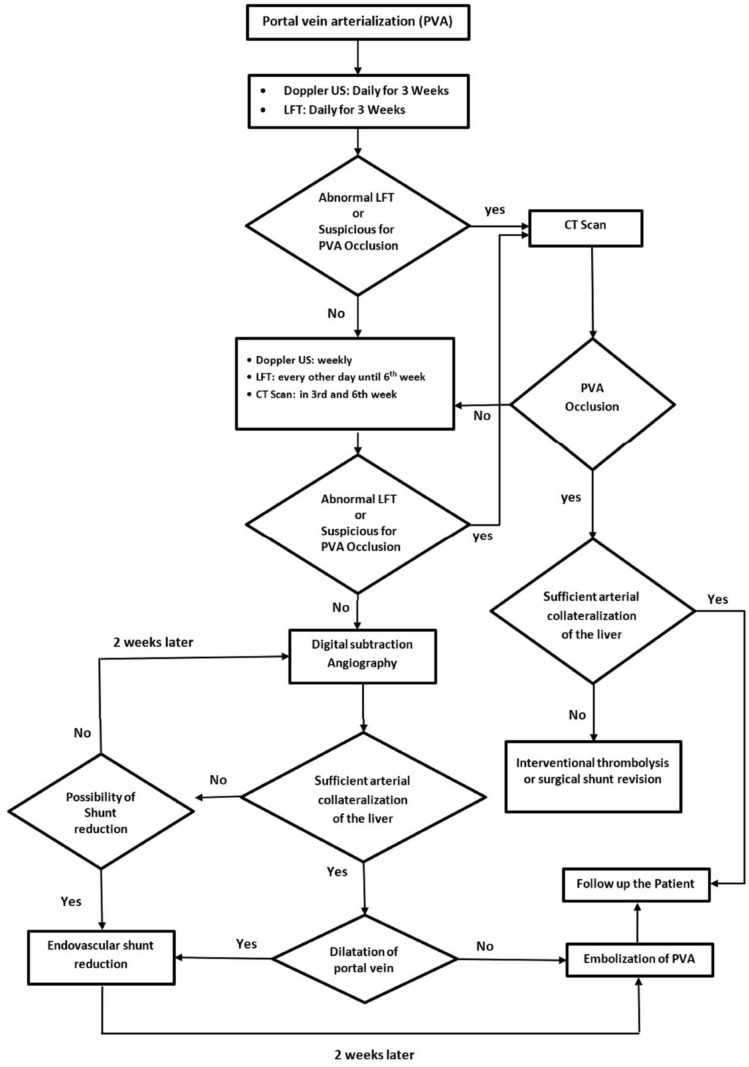
Standard operating procedures and flowchart guidelines for the postoperative management of patients undergoing portal vein arterialization.

Our clinical experience and a systematic review published by our team [7] were used to develop a first version of an interdisciplinary SOP for the postoperative management of a consecutive cohort of patients undergoing PVA. We modified the SOP based on the clinical outcomes of the patients and redesigned the SOP in a flowchart format as a postoperative management guideline (Figure 1). Since January 2019, we have been implementing the primary SOP for managing patients undergoing PVA at our center. The study protocol was approved by the independent ethics committee. 

According to our modified SOP, all patients undergoing PVA during HPB surgery are advised to undergo daily laboratory assessments of their liver function and Doppler ultrasonography of the liver for 3 weeks following PVA to monitor the patency of the arterioportal shunt and to rule out signs of portal hypertension.

Weekly Doppler ultrasonography and liver function assessments every other day are advised up to the 6th week after PVA. Any signs of liver damage or PVA shunt occlusion during this 6 week follow-up indicate the need for urgent computed tomography angiography (CTA) in the arterial and portal venous phases to assess whether interventional thrombolysis or operative shunt revision is required. Anticoagulant treatment was contemplated for all patients within the initial 6 weeks.

Routine CTA is performed in the 3rd and 6th weeks after PVA to assess the development of arterial collaterals to the liver and signs of portal hypertension.

Patients who have an uneventful 6 week follow-up undergo angiography to check whether endovascular embolization of the PVA is feasible. Initially, super-selective catheterization of the visceral arteries (i.e., coeliac trunk, superior mesenteric artery, and phrenic artery) is performed to assess liver perfusion. To enhance the evaluation of liver perfusion, an additional cone-beam CT can be performed.

**Figure 2:**
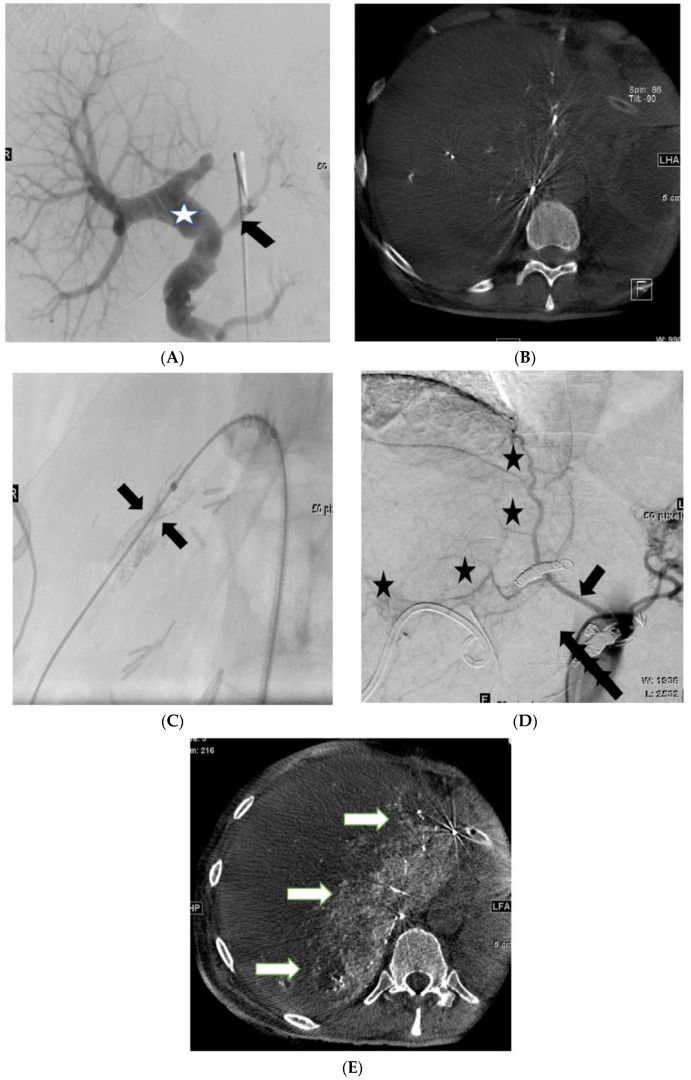
Two-stage arterioportal shunt embolization. (**A**): Angiography at the 6th postoperative week shows a patent arterioportal shunt (arrow) and moderate dilatation of the portal vein (asterisk). (**B**): 3D rotational angiography demonstrates inadequate arterial collateralization of the liver. (**C**): Shunt reduction with an hourglass-shaped balloon-expandable stent graft (arrows), proximal/distal diameter of 6 mm; neck diameter of 4 mm. (**D**): Detection of newly formed collateral arteries (asterisk) originating from the right inferior phrenic artery at the 8th postoperative week (arrow). (**E**): Super-selective catheterization of the right inferior phrenic artery followed by 3D rotational angiography shows a significant increase in arterial hepatic perfusion (arrows).

Patients with low arterial inflow (perfused liver volume: 0–50%) undergo an endovascular shunt reduction procedure to promote the further development of arterial collaterals. Follow-up angiography is performed 2 weeks later to assess the extent of collateral formation and determine whether shunt closure is feasible (Figure 2). The reduction of the caliber of the shunt entails the off-label use of commercially available tapered stents or partially expanded balloon-expandable stent grafts to induce flow reduction through the PVA [22]. Following the intervention, full heparinization is maintained until final embolization of the PVA. 

The same approach is employed for patients with arterial perfusion covering >50% of the liver but experiencing massive PV dilation. In these cases, an initial shunt reduction is performed before the PVA is definitively closed after 2 weeks. Moreover, full heparinization is maintained until final embolization of the PVA.

In contrast, patients with moderate-to-high arterial inflow (perfused liver volume > 50%) and no significant portal vein dilation are promptly scheduled for immediate embolization of the PVA using coils or plugs.

Patients are followed up after 1 and 5 years according to an individualized approach for evaluating the possible occurrence of portal hypertension and its complications.

### Patient Information

Early spontaneous occlusion of the PVA occurred one week after surgery due to inadequate arterial collateralization of the liver and was treated with interventional thrombolysis. Unfortunately, the patient died nine months later due to multiorgan failure. A late spontaneous occlusion of the PVA occurred in another patient six weeks after surgery. Given the satisfactory arterial collateralization of the liver, the patient was followed up regularly and had an uneventful postoperative period (41 months). One-stage PVA embolization was performed twelve weeks after surgery, and the patient remained uneventful during nineteen months of follow-up. Two-stage PVA embolization was performed six and eight weeks postoperatively in a case of portal vein dilatation. There were no complications at the 28 month follow-up.

## 3. Discussion

In this study, we presented an SOP for the postoperative management of patients undergoing PVA following the total de-arterialization of the liver. This SOP, developed based on a systematic review [7] and our institutional experience spanning 4 years [23], is tailored to guide the care of these patients. Our goal was to emphasize the relevance of this approach to interventional radiologists, who play a pivotal role in the post-PVA care process. 

PVA has shown promising outcomes in both liver transplantation and hepatic resection, with an overall survival rate of 63% at a median follow-up duration of 13 months [5]. However, PVA is associated with high morbidity rates. Complications related to portal hypertension, such as refractory ascites, intra-abdominal or variceal bleeding, insufficient inflow leading to PVA shunt thrombosis, and aneurysmal dilatation of the portal branches, are among the adverse effects of PVA [24]. Arteriovenous shunt-related complications, such as thrombosis, can necessitate emergency liver transplantation [25,26,27,28]. Although delayed occlusion is often well tolerated, early thrombosis can be catastrophic, leading to acute liver ischemia [21]. Therefore, postoperative surveillance is crucial to manage or prevent the onset of portal hypertension, highlighting the need for a standardized postoperative guideline.

Patients undergoing PVA typically experience a rapid rise in the levels of aspartate aminotransferase, alanine aminotransferase, lactate dehydrogenase, and bilirubin during the first 3 postoperative days. These values gradually decrease until discharge and return to their normal ranges after 2 months [29]. Therefore, we recommend daily measurement of liver function in the first 3 postoperative weeks and every other day between the 3rd and 6th postoperative weeks.

Thrombosis, which frequently develops in the anastomoses of the celiac trunk branches, is the most prevalent early complication, affecting 18% of patients undergoing PVA [7]. This complication can be identified in the early stages after the procedure using routine Doppler ultrasonography [5]. In this modified PVA-SOP, we recommend daily Doppler ultrasonography in the first 3 postoperative weeks and weekly ultrasonography between the 3rd and 6th postoperative weeks. This monitoring is essential to assess hepatic perfusion and detect any indications of portal hypertension.

The management of PVA-related thrombosis involves the use of anticoagulants and, in some cases, reoperation [5,30]. According to Bhangui et al., no further intervention is required in the presence of thrombosis if collateral vessels are present; however, if arterial collateral flow is absent, a second PVA procedure should be performed [5]. This recommendation aligns with our PVA-SOP. Considering an endovascular intervention aimed at reopening the blocked vessels through local fibrinolysis and/or aspiration thrombectomy is also possible.

A significant challenge in PVA is determining the optimal timing for shunt closure. Shunt closure should ideally occur after adequate arterial collateralization has developed but before the onset of portal hypertension. Deliberate and attentive CTA examination in the 3rd and 6th postoperative weeks plays a pivotal role in this decision-making process. CTA allows us to assess the presence of portal hypertension and provides an initial estimation of the extent of collateral inflow to the liver.

Following our SOP, patients displaying signs of arterial collateralization on CTA after 6 weeks proceed to the next step, which involves conventional angiography.

Through super-selective catheterization of the visceral vessels and the use of 3D rotational angiography (3DRA), we can highly and precisely understand the extent of liver segments perfused by arteries (Figure 2). It is important to emphasize that the threshold of 50% used to determine whether a patient undergoes PVA embolization is empirically based because no existing literature has specified the precise perfusion level at which safe closure of the arterioportal fistula is feasible. In addition to the purely volumetric perfusion approach, considering the patient’s other cardiovascular and metabolic comorbidities is imperative.

**Figure 3:**
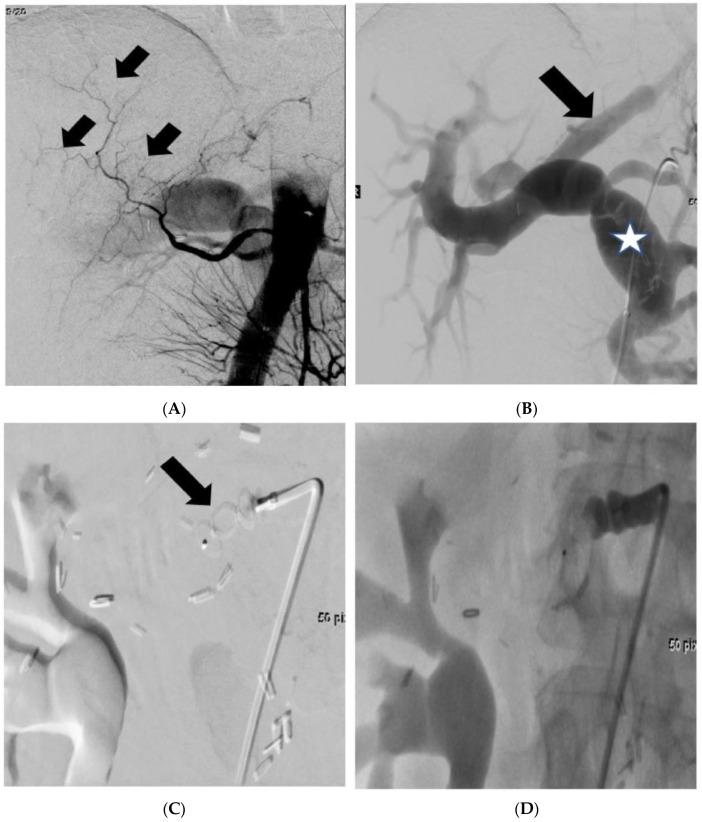
One-stage arteriovenous shunt embolization. (**A**): Digital subtraction angiography shows considerable hepatic perfusion by well-developed collateral inflow (arrows) arising from the superior mesenteric artery. (**B**): Pre-embolization view: patent arteriovenous shunt (arrow). Note: massive dilatation of the portal vein (asterisk). (**C**): Placement of an 8 mm vascular plug (arrow) via a 5-French sheath in front of the anastomosis of the arteriovenous shunt. (**D**): Post-embolization view: complete closure of the arteriovenous shunt without remaining inflow to the portal vein. Please note that this patient experienced portal venous thrombosis 2 days after shunt closure (not visible).

The introduction of endovascular shunt reduction to regulate inflow in preparation for final shunt embolization after 2 weeks was incorporated into our practice based on cases where patients developed complete PV thrombosis following shunt occlusion. These patients exhibited signs of portal hypertension and substantial portal vein dilatation before PVA closure (Figure 3). Therefore, we hypothesized that the sudden pressure relief resulting from the closure of the arterioportal fistula, particularly in the presence of a previously massively dilated PV, may have contributed to the further formation of thrombi in these instances. Therefore, meticulous post-closure observation is imperative for these patients, including daily duplex sonography to assess vascular patency and a 1 week course of full heparinization.

In addition to avoiding a sudden pressure drop, the use of subsequent shunt reduction may also help prevent liver over-arterialization or the development of further collaterals in patients with previously inadequate arterial inflow in imaging studies.

Utilizing this SOP offers numerous benefits, such as precise timing for closing the PVA to potentially avert portal hypertension. The implementation of this SOP has enhanced the practicality of the technique with greater efficacy, resulting in lower morbidity and mortality compared to the literature [7,23]. This is achieved by mitigating the risk of ischemic cholangiopathy that can result from early PVA occlusion.

A potential limitation of this study is the absence of a cohort representing the efficacy of the current SOP. Ongoing studies aim to validate the reliability of the proposed SOP across a larger and more diverse group of healthcare professionals. In conclusion, we have introduced a promising SOP for the postoperative management of patients undergoing PVA during HPB surgeries. This SOP simplifies the postoperative period, making it manageable for various healthcare professionals with varying levels of experience in hepatic surgery, including interventional radiologists. The PVA-SOP proposed in this study could be used to tailor the postoperative management of each patient. Further experimental studies and larger case series with longer follow-up durations are required to evaluate the reproducibility of this SOP.

The recommendations and considerations outlined here represent a modified treatment algorithm in the form of an institutional SOP, integrating both the single-center experience and findings from a thorough literature review conducted by the authors of this manuscript. Our working group remains committed to updating and refining the SOP as additional data and insights emerge, ensuring its ongoing alignment with best practices in our setting. 

## 4. Conclusions

In this study, we introduced a modified standard SOP for managing patients after PVA following total de-arterialization of the liver. This SOP aims to reduce complications and improve patient outcomes by providing clear, consistent guidelines for interventional radiologists and surgeons, emphasizing routine monitoring and optimal timing for interventions.

## Data Availability

Not applicable.
